# Screening of a New *Kosakonia* Species for Polyethylene Biodegradation

**DOI:** 10.4014/jmb.2411.11058

**Published:** 2025-03-13

**Authors:** Jin-Hee Cho, Seung-Do Yun, Hyun-Woo Kim, Min-Ju Seo, Bong Hyun Sung, Soo-Jin Yeom

**Affiliations:** 1 School of Biological Sciences and Biotechnology, Graduate School, Chonnam National University, Gwangju 61186, Republic of Korea; 2 Institute of Synthetic Biology for Carbon Neutralization, Chonnam National University, Gwangju 61186, Republic of Korea; 3 Synthetic Biology Research Center, Korea Research Institute of Bioscience and Biotechnology, Daejeon 34141, Republic of Korea; 4 School of Biological Sciences and Technology, Chonnam National University, Gwangju 61186, Republic of Korea; 5 Institute of Systems Biology and Life Science Informatics, Chonnam National University, Gwangju 61186, Republic of Korea

**Keywords:** Polyethylene, biodegradation, *Kosakonia cowanii*

## Abstract

Polyethylene (PE) is among the most widely used synthetic plastics globally, serving as an essential material in daily life and numerous industries, such as packaging for bottles and food, as well as in the production of toys and pipes. PE is used for various purposes owing to its high durability and low production costs, leading to a steadily increasing demand. However, PE waste is a significant contributor to environmental pollution, posing serious threats to marine and soil ecosystems. Therefore, the efficient decomposition of PE, a synthetic polymer known for its resistance to degradation, using bacteria offers a sustainable and effective method for reusing PE. In this study, we isolated a novel species of *Kosakonia*, designated *Kosakonia cowanii* JNU01, from a landfill site, capable of biodegrading PE. *K. cowanii* JNU01 exhibited the highest cell growth rate in media containing PE, indicating its effectiveness in decomposing PE for use as a sole carbon source in its metabolic pathway. Treatment of PE with *K. cowanii* JNU01 resulted in the emergence of new chemical functional groups, including hydroxyl, carboxyl, amide, and ether groups, within the inert hydrocarbon structure. Analysis of the PE film treated with *K. cowanii* JNU01 revealed considerable physical degradation on the film’s surface. Additionally, various metabolites released from PE by *K. cowanii* JNU01 were identified. These findings suggest that *K. cowanii* JNU01 proves to be an effective candidate bacterium for PE degradation.

## Introduction

With the increasing use of plastics, production and waste generation have risen correspondingly [1]. This has led to the accumulation of plastic in landfills and natural environments, posing serious threats to ecosystems and causing harmful impacts on human health and numerous wildlife species. To address these issues, various preliminary studies and interventions have been undertaken, including recycling, thermal degradation, and biological degradation [2, 3]. Among these approaches, biological degradation is gaining attention as an economically viable and environmentally friendly solution for plastic waste reduction [4, 5]. Traditional plastic waste treatment methods such as incineration, release organic pollutants, particulate matter, and hazardous substances like dioxins and furans into the environment [6-9]. Therefore, the biodegradation of plastics using biocatalysts such as bacteria and their enzymes represents an ideal solution to mitigate adverse environmental effects [10].

The backbone of PE consists solely of C-C single bonds, lacking functional groups, and its structural stability grants it significant resistance to decomposition [11]. PE materials, such as low-density PE (LDPE) and high-density PE (HDPE), are the most widely used petroleum-based polymers for manufacturing disposable plastic items, accounting for up to 64% of disposable plastics. LDPE, in particular, is discarded within a short period after use, leading to its rapid accumulation in the environment [3, 12, 13]. Therefore, it is imperative to identify an eco-friendly and sustainable method to address this issue [3, 12]. Recent studies have isolated several PE-degrading bacteria from various environments: *Rhodococcus ruber* from agricultural waste in soil [14-16]; *Klebsiella pneumoniae* from soil [17]; *Desulfotomaculum nigrificans* and *Pseudomonas alcaligenes* from plastic waste contaminated soil [18]; *Stenotrophomonas* sp., *Comamonas* sp., and *Delftia* sp. from plastic debris in soil [19]; *Enterobacter* sp. D1 from the gut of *Galleria mellonella* [20]; *Pseudomonas putida* from garden soil [21]; as well as various PE-degrading bacteria (namely, *Bacillus megaterium*, *B. thuringiensis*, *B. cereus*, *B. amylolyticus*, *B. subtilis*, *Pseudomonas putida*, and *P. fluorescens*) from soil [22-24]. Enzymes reported to be involved in PE biodegradation include alkane-1-monooxygenase [25, 26], laccase-like multi-copper oxidase [17, 27], and cytochrome P450 [24], and PEase [28]. Although bacteria capable of degrading PE and certain enzymes predicted to be involved in PE degradation have been identified, the precise mechanism underlying PE degradation remains unclear. Therefore, further investigation to elucidate this mechanism is essential, and discovering biodegradable biocatalysts capable of breaking down PE is a critical step. Screening for promising bacteria with PE-degrading capabilities is necessary to identify additional biocatalysts involved in PE biodegradation.

In this study, we identified promising new microbial candidates for PE degradation. Specifically, we successfully isolated a PE-degrading strain, *Kosakonia cowanii* JNU01, from the Metropolitan City Sanitary Landfill in Gwangju, Korea. The strain demonstrated the ability to grow using PE as its sole carbon source. We confirmed the PE degradation ability of *K. cowanii* JNU01 by characterizing the chemical and physical changes in PE through Fourier transform infrared spectroscopy (FT-IR) and scanning electron microscopy (SEM) analyses. Additionally, we analyzed the metabolites released during incubation with PE and *K. cowanii* JNU01 using gas chromatography-mass spectrometry (GC-MS). The successful screening of *K. cowanii* JNU01 as a PE-degrading bacterium lays the groundwork for identifying additional PE-degrading enzymes and elucidating the mechanisms underlying PE biodegradation.

## Material and Methods

### Chemicals Used in the Study

This study utilized M9 minimal broth (6.0 g/l Na_2_HPO_4_, 3.0 g/l KH_2_PO_4_, 0.5 g/l NaCl, and 1.0 g/l NH_4_Cl, 240.7 mg/l MgSO_4_, 11.098 mg/l CaCl_2_) along with a trace element solution (21.8 mg/l CoCl_2_·6H_2_O, 21.6 mg/l NiCl_2_·6H_2_O, 24.6 mg/l CuSO_4_·5H_2_O, 1.62 g/l FeCl_3_·6H_2_O, 0.78 g/l CaCl_2_, and 14.7 mg/l MnCl_2_·4H_2_O) were used. Luria-Bertani (LB) medium was procured from MB Cell (Republic of Korea), and methanol (≥99.8%) and heptane (≥98%) were purchased from Duksan (Republic of Korea). Chloroform (≥99.5%) and ethyl acetate (≥99.5%) were sourced from Daejung (Republic of Korea). Additionally, PE, with an average M_w_ (Weight-average molecular weight) of approximately 4,000 and an average M_n_ (Number-average molecular weight) of approximately 1,700, possessing typical physical properties of LDPE, was purchased from Sigma-Aldrich (USA). To improve the resolution of PE, the external surface area was increased through a reprecipitation process. The re-precipitation process for PE powder and film was the same as the method described in our previous study [24].

### Identification of PE Biodegradable Bacterial Strains

Environmental samples, including soil and plastic waste, were collected from the Metropolitan City Sanitary Landfill located in Gwangju, Korea. The sample (50 g) was mixed with 450 ml of phosphate-buffered saline (PBS) and then filtered using Whatman paper with an 11 μm pore size. The filtered solutions were spread onto liquid M9 minimal broth agar plates supplemented with 1 g/l PE powder and incubated at 28°C for 7 days. Bacterial colonies that developed were re-streaked on M9 minimal broth agar plates containing 1 g/l PE. The cultured bacteria were subsequently incubated in liquid M9 minimal broth containing 1 g/l PE powder at 28°C with shaking at 150 rpm for 7 days. To evaluate bacterial growth, the optical density (OD_600_) of 200 μl of culture solution was measured on day 0 and day 7 using a VICTOR Nivo multimode microplate reader (VICTOR Nivo Multimode Microplate Reader, PerkinElmer, USA). The fold-change was calculated by dividing the OD_600_ reading on day 7 by the OD_600_ reading on day 0. Colony #2 was streaked onto LB agar plates and incubated overnight at 28°C. Subsequently, DNA extraction and 16S rRNA sequencing were conducted following the method established in our previous study [24]. In addition to the 16S rRNA sequences of the newly identified *Kosakonia cowanii* JNU01, sequences from nine other *Kosakonia* species, three *Enterobacter* species, and *Vibrio aerogenes* were obtained from the NCBI nucleotide database. Phylogenetic trees were constructed using the maximum-likelihood (ML) method with the Tamura-Nei model and 1,000 bootstrap replicates in MEGA11 (version 11.0.13) software [29].

### Growth Curve and Dissolved Oxygen Analysis of *K. cowanii* JNU01 in PE Media

*K. cowanii* JNU01 was first cultured in LB media and incubated overnight at 28°C with shaking at 200 rpm. The culture was then centrifuged at 4,000 rpm for 20 min, and the supernatant was removed. The resulting bacterial pellet was washed with M9 minimal broth before being inoculated into M9 minimal broth containing PE powder at concentrations of 10, 30, and 50 mg/l, respectively, supplemented with 0.1% trace element solution. The cultures were incubated at 28°C with shaking at 200 rpm for 35 days. The initial OD_600_ was 0.2, and the experiment included two controls: one containing only PE powder in M9 media and the other containing bacteria without PE powder. Every five days, 500 μl of culture and controls were diluted with 500 μl of fresh M9 broth, and OD_600_ was measured using a UV-Vis spectrophotometer (UV-1900, Shimadzu, Japan).

Additionally, dissolved oxygen (DO) concentration in M9 minimal broth was monitored using a biochemical oxygen demand (BOD) probe and a DO meter (YSI 4100 BOD IDS PROBE & YSI 4010-1W DO meter, YSI Incorporated, USA). The DO measurements were conducted at an initial OD_600_ of 0.2 in 60 ml BOD bottles containing PE at a concentration of 30 mg/ml, with incubation at 28°C and shaking at 140 rpm for 4 days.

### GC-MS Analysis of PE Media after Treatment of *K. cowanii* JNU01

The cultured solution was filtered using Whatman filter paper with an 11 μm pore size, and any remaining PE particles were rinsed with methanol. The filtered culture solution was then combined with ethyl acetate in a 1:1 ratio, vortexed for 10 min, and centrifuged at 13,000 rpm for 20 min to isolate the supernatant. The extract was analyzed using a gas chromatograph-mass spectrometer (GC-MS) (QP2020 NX, Shimadzu) equipped with an electron impact ionization source and a DB-5MS capillary column (Agilent, USA). The column temperature was programmed to increase from 40°C to 280°C at a rate of 6°C min^–1^, with an initial hold at 40°C for 2 min and a final hold at 280°C for 3 min. The chemical structures of the products were determined using the GC-MS data and the NIST/WILEY database.

### FT-IR Analysis of *K. cowanii* JNU01-Treated PE Powder

Residual PE particles rinsed with methanol were subjected to Fourier transform infrared (FT-IR) analysis to characterize alterations in the chemical structure of the PE polymer chains. FT-IR measurements (Spectrum Two, PerkinElmer) were conducted to identify the chemical functional groups in the PE samples across a wavenumber range of 4,000‒700 cm^–1^, with a resolution of 4 cm^–1^.

### SEM Analysis of *K. cowanii* JNU01-Treated PE Film

Scanning electron microscope (SEM) was employed to examine the physical properties of PE films. The surface morphologies of pure and biologically degraded PE films were observed using a field-emission SEM (FE-SEM; Hitachi S-4800, Japan) operating at an accelerating voltage of 15 kV (Center for Scientific Instrument, Chosun University, Gwangju, Korea). *K. cowanii* JNU01 was inoculated into a flask containing 10 ml of liquid M9 minimal broth supplemented with PE film and 0.1% trace element solution. With an initial OD_600_ of 2.0, *K. cowanii* JNU01 was cultured at 28°C and 80 rpm for 30 days. Following incubation, the biologically degraded and pure PE films were washed in a sodium dodecyl sulfate solution (SDS; 2%, w/v) for 6 h and subsequently rinsed twice with fresh methanol. This pretreatment procedure was employed to remove any bacterial cells adhering to the PE surface, allowing for accurate surface morphology assessment. To prepare the PE films for SEM imaging, a platinum (Pt) layer was sputtered at 20 mA for 60 s to generate a thin, conductive coating, thus facilitating clear top-view imaging of the non-conductive polymer samples.

## Results and Discussion

### Isolation and Selection of PE-Biodegrading Bacteria

**Fig. 1:**
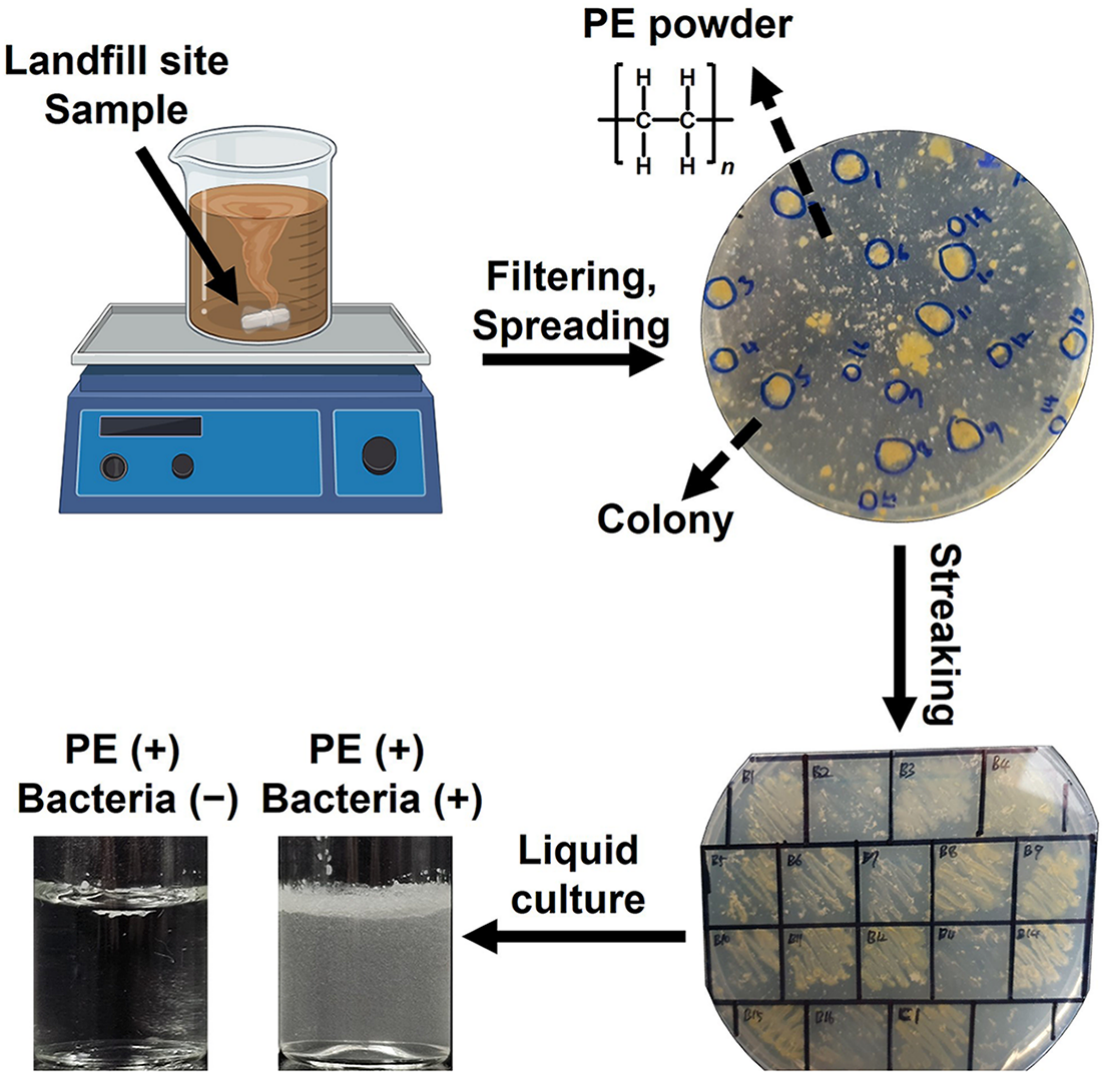
Overview of the screening process for PE-biodegrading microorganisms. The landfill site sample was obtained from the metropolitan city sanitary landfill in Gwangju, Korea.

**Fig. 2:**
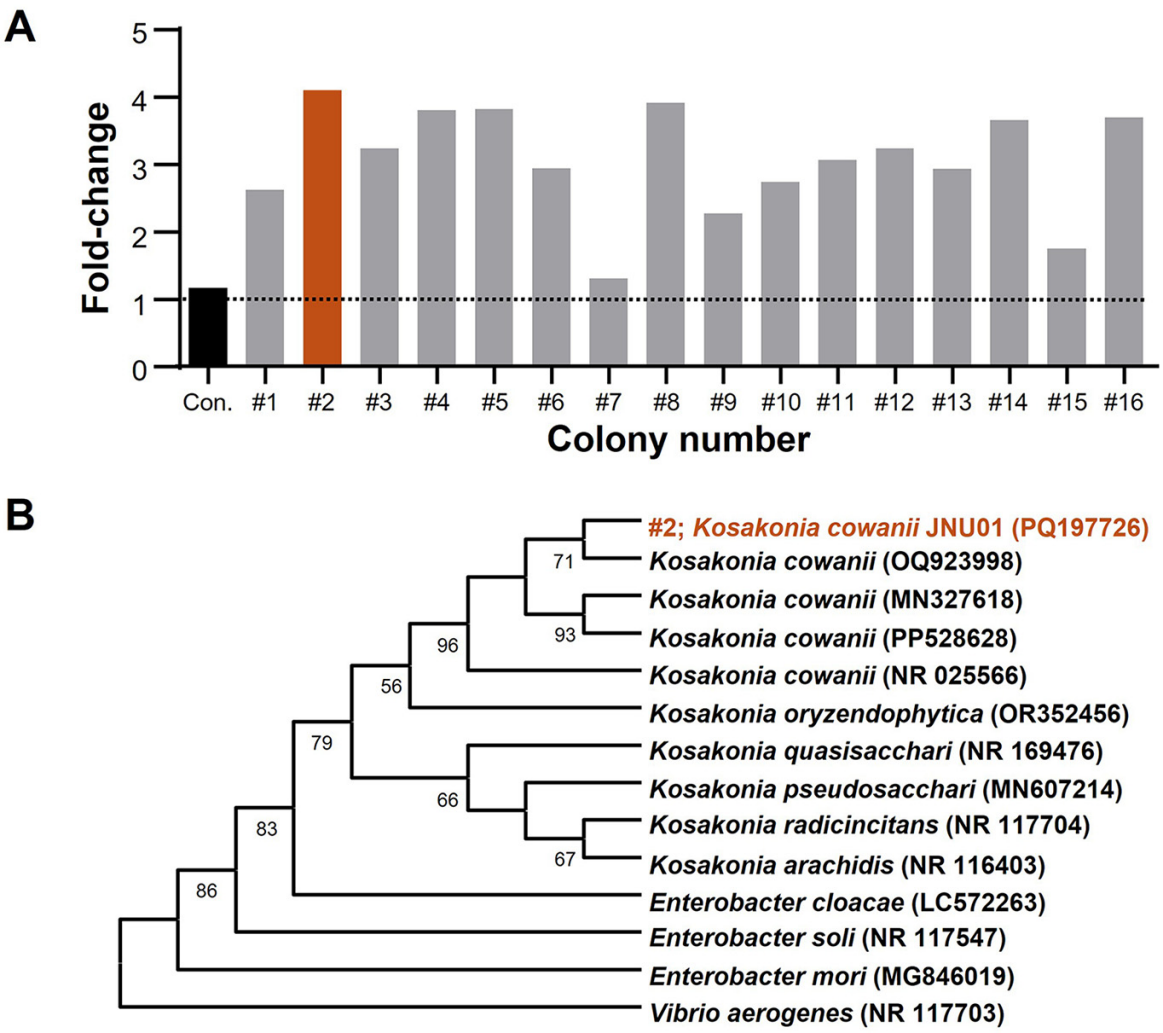
Identification process of PE-degrading microorganisms. (**A**) Fold-change in OD_600_ (Day 7 OD_600_/Day 0 OD_600_) for 16 individual colonies. (**B**) Phylogenetic tree of *K. cowanii* JNU01 based on 16S rRNA gene sequences, with accession numbers in parentheses.

Landfill soil samples were initially screened for PE-biodegrading bacteria, using PE powder as the sole carbon source (Fig. 1). Sixteen single colonies were obtained and cultured on M9 minimal broth agar plates supplemented with PE powder. These colonies were then grown in liquid M9 minimal broth containing only PE powder for 7 days. One bacterial species, identified as #2, exhibited a 4.1-fold increase in OD_600_ from day 0 to day 7, indicating significant growth (Fig. 2A). A total of 14 different 16S rRNA sequences were analyzed and compared with those of closely related species (Fig. 2B). The bacterial species #2 formed a distinct monophyletic clade with *K. cowanii*, leading us to designate this strain as #2 *K. cowanii* JNU01 (Fig. 2B). Common microorganisms known to degrade PE include *Acinetobacter*, *Alcanivorax*, *Bacillus*, *Brevibacillus*, *Cobetia*, *Comamonas*, *Delftia*, *Enterobacter*, *Halomonas*, *Klebsiella*, *Paenibacillus*, *Pseudomonas*, and *Rhodococcus* species [13, 30-32]. In this study, *K. cowanii* JNU01 is identified as a novel PE-degrading bacterium with potential applications in PE degradation as well as in the decomposition of cotton textile waste [33].

### Growth of *K. cowanii* JNU01 in PE Media

**Fig. 3:**
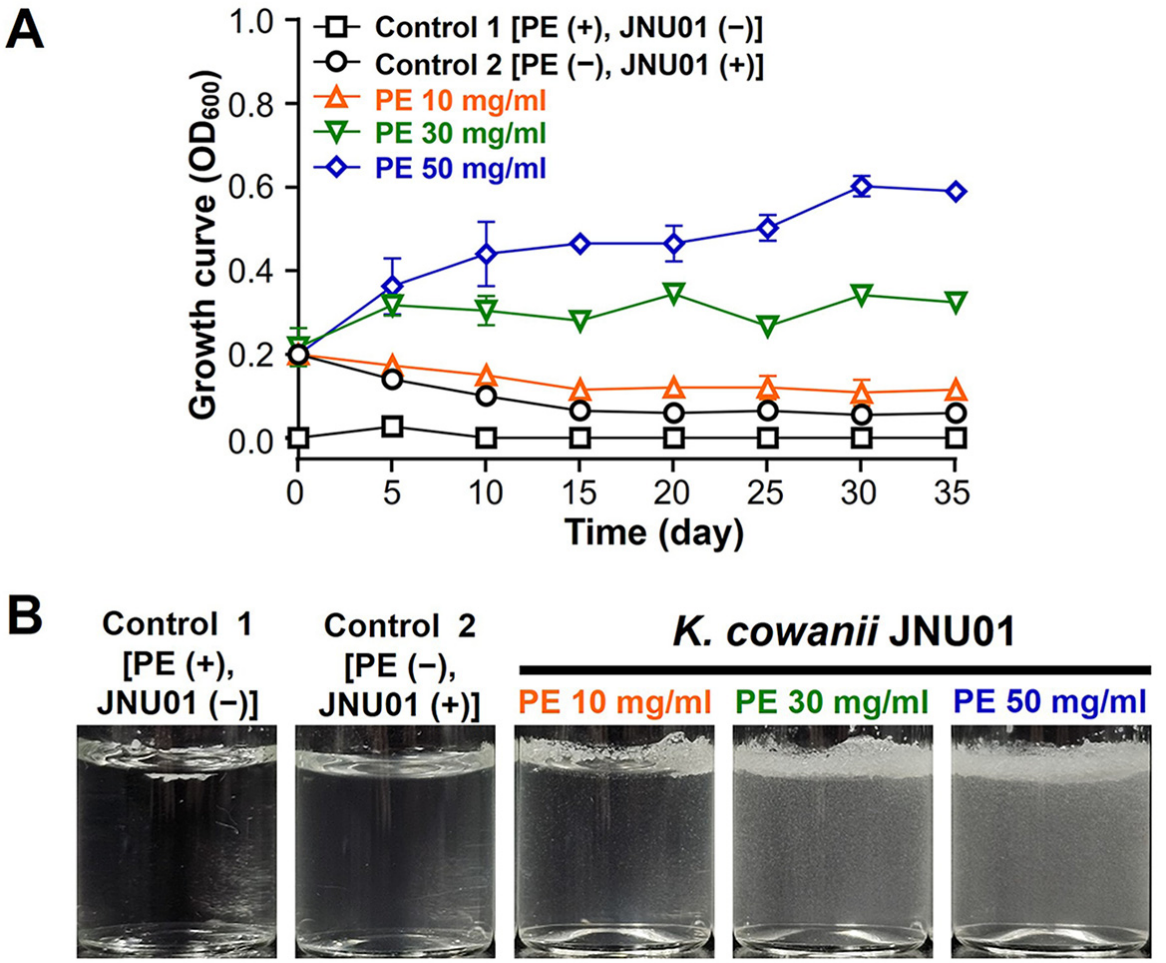
Growth curve of *K. cowanii* JNU01 in PE media. (**A**) Growth curve of *K. cowanii* JNU01 in PE media. The orange triangle, green inverted triangle, and blue diamond represent *K. cowanii* JNU01 inoculated with 10, 30, and 50 mg/ml of PE powder, respectively. (**B**) Image of medias with *K. cowanii* JNU01 and PE powder. Control 1 represents a sample containing only PE powder, and Control 2 contains only *K. cowanii* JNU01.

The growth curve of *K. cowanii* JNU01 was determined by culturing the bacterium in M9 minimal broth medium using PE powder as the sole carbon source at concentrations of 10, 30, and 50 mg/ml for 35 days at 28°C. Significant increases in turbidity were observed at higher PE concentrations (Fig. 3A). At 10 mg/ml PE, the OD_600_ value remained constant, indicating no significant growth in the absence of microbes. However, as the PE concentration increased to 30 and 50 mg/ml, the OD_600_ values rose markedly. Notably, *K. cowanii* JNU01 achieved a very high OD_600_ value of 0.44 within just 10 days at 50 mg/ml PE, demonstrating its exceptional capacity to grow using PE as a carbon source over a 35-day incubation period (Fig. 3A). According to previous studies summarized in Table 1, *Enterobacter* sp. D1 was cultured at 37°C using PE film as the sole carbon source, achieving an OD_600_ value of 0.24 after 31 days [20]. Similarly, a consortium of *Acinetobacter* sp. NyZ450 and *Bacillus* sp. NyZ451 reached an OD_600_ value of 0.065 after 30 days at 28°C using PE particles as the carbon source [34]. Furthermore, *Pseudomonas aeruginosa* SKN1 achieved an OD_600_ value of approximately 0.24 after 60 days at 29°C using PE powder [35]. These results suggest that *K. cowanii* JNU01 can utilize PE as the sole carbon source independently, without forming a consortium, and achieve rapid and efficient growth under relatively moderate temperature conditions (28°C) within a short period. Additionally, the PE powder cultured with *K. cowanii* JNU01 exhibited a tendency to blend into the PE media, indicating that the PE surface becomes more hydrophilic as it is colonized by microorganisms (Fig. 3B).

This hydrophilicity likely facilitates further colonization and enhances microbial activity on the hydrophilized PE surface [36-40]. To evaluate the PE mineralization by *K. cowanii* JNU01, dissolved oxygen (DO) was measured. The initial DO was 8.93 ± 0.01, decreasing to 2.79 ± 0.01 after 1 day, 0.54 ± 0.01 after 2 days, and 0.27 ± 0.10 on the 4th day (Table 2). Overall, these results provide additional evidence of PE mineralization [41], suggesting that *K. cowanii* JNU01 can effectively utilize PE as a carbon source.

### Identification of Metabolites in Culture Media with PE

**Fig. 4:**
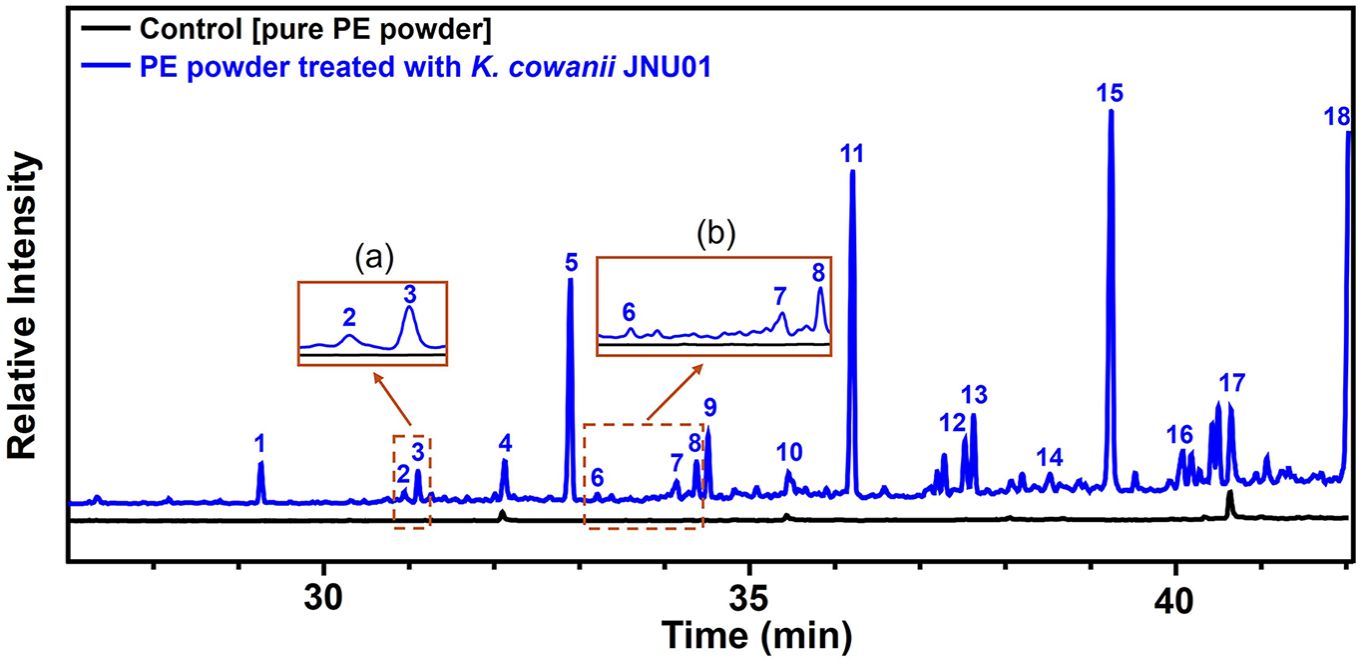
GC-MS analysis of PE culture media inoculated with *K. cowanii* JNU01. Control is a sample containing only PE powder, and the experimental group includes PE powder treated with *K. cowanii* JNU01. (a) Magnified regions corresponding to peaks #2 and #3 are shown. (b) Magnified regions corresponding to peaks #6, #7, and #8, shown in the inset boxes.

To identify metabolites produced when PE is used as a carbon source, the culture medium with *K. cowanii* JNU01 and added PE powder was analyzed using GC-MS. The results revealed the formation of alkanes, ketones and fatty acids (Fig. 4 and Table 3). Using the NIST/WILEY database, GC-MS analysis of *K. cowanii* JNU01 identified a variety of metabolites, including ketone compounds with carbon chain lengths ranging from C_16_ to C_33_. Key compounds detected included 2-hexadecanone, 9-heptadecanone, 2-heptadecanone, 9-octadecanone, 2-nonadecanone, 10-nonadecanone, 2-nonacosanone, and 2-tritriacontanone. In addition, alkanes such as octadecane, eicosane, pentacosane, 2-methylhexacosane, and dotriacontane, along with several oxidized compounds, were also identified as metabolites (Table 3). Compared to the findings from previously reported studies summarized in Table 1, *K. cowanii* JNU01 exhibits distinct characteristics in its metabolic profile. For example, metabolites detected in *Enterobacter* sp. D1 primarily included alkanes, alcohols, hydrocarbons, esters, and acids with carbon chain lengths below C_20_ [20]. Metabolites in *Klebsiella pneumoniae* CH001 were identified as carboxylic acids ranging from C_2_ to C_18_ and alkanes from C_2_ to C_19_ [42]. *Bacillus velezensis* C_5_ was reported to have n-alkanes identified within the range of C_24_ to C_29_ [43]. While metabolites, including alkanes and alcohols with chain lengths spanning from C_14_ to C_36_, were detected in a consortium of *B. cereus*, *B. megaterium*, and *B. subtilis* [23]. Additionally, the PE metabolites derived from *B. pumilus*, *B. halodenitrificans*, and *B. cereus* included ketone compounds with carbon chains below C_16_, alkanols below C_20_, and alkanes below C_30_ [44]. These findings suggest that *K. cowanii* JNU01 metabolizes PE, resulting in the production of fatty acids, alkanes, and long-chain ketone compounds. The alkanes, ketones, and fatty acids produced during the PE degradation process by *K. cowanii* JNU01 are consistent with the previously proposed PE degradation pathway [10]. In this pathway, PE is initially hydroxylated at subterminal and in-chain carbons by hydroxylases such as cytochrome P450 (CYP)[24], unspecific peroxygenase (UPO), and alkane monooxygenase (AlkB). The resulting alcohols are further oxidized to ketones via alcohol dehydrogenase (Adh), producing metabolites such as 2-hexadecanone, 9-heptadecanone, 2-heptadecanone, 9-octadecanone, 2-nonadecanone, 10-nonadecanone, 2-nonacosanone, and 2-tritriacontanone. In next step, Baeyer–Villiger monooxygenase (BVMO) catalyzes the insertion of an oxygen atom into the carbonyl group of the intermediate ketone, leading to the formation of an ester group which are hydrolyzed by esterase to form alkanoic acids (*e.g.*, octadecanoic acid) and alcohols. Additionally, alkanols further oxidized to ketones and carboxylic acids via Adh and aldehyde dehydrogenase (Aldh). The metabolites observed in this study show a notable correlation with the proposed PE degradation pathway. To further elucidate the enzymatic and metabolic networks involved in PE degradation, a whole genome analysis of *K. cowanii* JNU01 should be performed. This analysis could help identify key genes and enzymes responsible for mediating PE breakdown, providing valuable insights for optimizing microbial strategies to enhance PE degradation.

### Functional Group Changes of PE Treated with *K. cowanii* JNU01

**Fig. 5:**
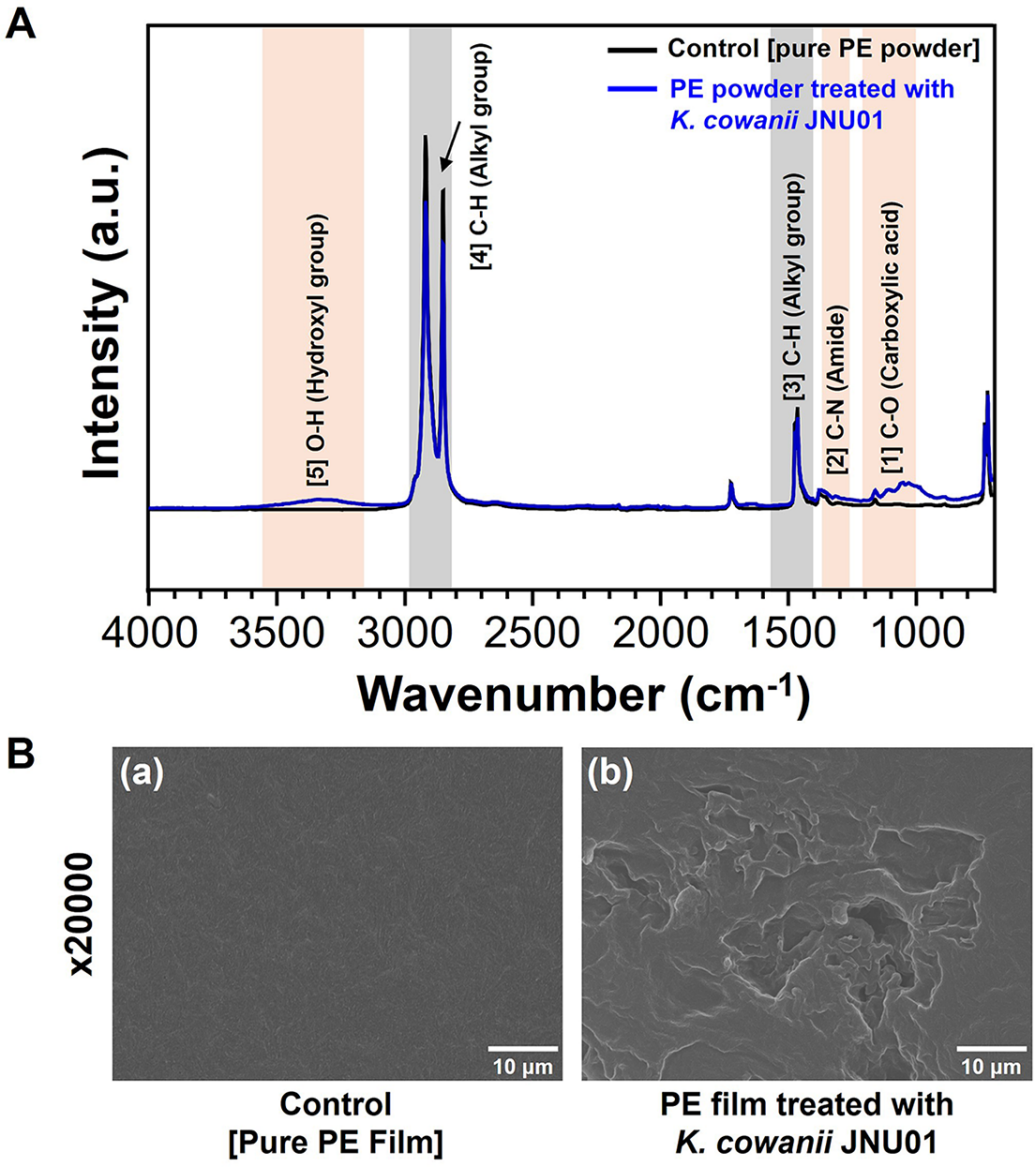
FT-IR and SEM analyses of PE powder and PE film treated with *K. cowanii* JNU01. (**A**) FT-IR analysis of PE powder treated with *K. cowanii* JNU01. [1] C–O (carboxylic acid), [2] C–N (amide), [3] C–H (alkyl group), [4] C–H (alkyl group), [5] O–H (hydroxyl group). (**B**) SEM analysis of *K. cowanii* JNU01 treated PE film: (a) The control (pure PE film) sample, and (b) The PE film treated with *K. cowanii* JNU01.

The newly formed chemical functional groups in PE powder treated with *K. cowanii* JNU01 were analyzed using FT-IR spectroscopy (Fig. 5A and Table 4). PE samples incubated with *K. cowanii* JNU01 exhibited increased characteristic peaks corresponding to hydroxyl groups (O–H; 3,200–3,400 cm^–1^), carboxylic acids (C–O; 1,000–1,320 cm^–1^), and amides (C–N; 1,200–1,350 cm^–1^), compared to the control sample. These changes indicate the formation of oxygen- and nitrogen-rich functional groups through the biodegradation of PE powder [24, 34, 45, 46]. Notably, the decrease in characteristic peaks associated with alkyl groups (C–H; 2,850–3,000 and 1,445–1,485 cm^-1^) can be attributed to the consumption of alkane chains [47]. This finding suggests the existence of multiple biodegradation pathways for PE, including the formation of oxygen-rich functional groups by *K. cowanii* JNU01. Hydroxylation of the C–C bonds in PE is a key step in the biodegradation mechanism previously proposed [10]. FT-IR analysis of *K. cowanii* JNU01-treated PE indicates that degradation occurred on the surface of the PE powder following treatment with the *Kosakonia* strain. In summary, our study demonstrates that *K. cowanii* JNU01-biodegraded PE exhibits diverse chemical functionalities, including hydroxyl groups, carboxylic acids, and amides, highlighting the various chemical transformations that occur during the biodegradation process.

### Observation of Surface Changes in PE Film Treated with *K. cowanii* JNU01

The surface morphology of pure PE film and PE film biologically degraded by *K. cowanii* JNU01 after one month of incubation was examined using FE-SEM to assess the external morphological degradation of PE. The untreated PE film displayed a smooth surface, free of any defects such as holes or cracks (Fig. 5B (left, a)). In contrast, the *K. cowanii* JNU01-treated PE film exhibited numerous surface defects (Fig. 5B (right, b)), including micron-sized pores with a broad size distribution. These observations align with findings from previous studies on PE biodegradation by various bacterial strains, such as *Enterobacter* sp. D1, *Acinetobacter* sp. NyZ450, *Bacillus* sp. NyZ451, *Exiguobacterium* sp., *Ochrobactrum* sp., *Halomonas* sp., *B. amyloliquefaciens* BSM-1, *B. amyloliquefaciens* BSM-2, *Enterobacter asburiae* YT1, and *Bacillus* sp. YP1 [20, 34, 48-50]. Consequently, it was confirmed that *K. cowanii* JNU01 is potential PE decomposer. And then it can be used to generate new synthetic plastic-eating bacteria by introducing genome editing and providing new pathways for PE upcycling.

## Conclusion

Environmental pollution caused by plastics poses a significant threat to ecosystems and human health, making it a critical issue to address. In this study, we isolated a novel *Kosakonia* strain from landfill soil capable of biodegrading PE. The strain, *K. cowanii* JNU01, demonstrated growth using PE as its sole carbon source, reaching an OD_600_ value of 0.62 at a PE concentration of 50 mg/ml. Observations of morphological changes on the PE surface, the detection of various new functional groups, and the release of degradation by-products strongly support the PE degradation capability of *K. cowanii* JNU01. Furthermore, various alkanones released following the PE biodegradation by *K. cowanii* JNU01 offer potential as renewable resources for producing high-value products. Thus, bacterial PE biodegradation represents a promising approach to plastic-refinery, which could transform plastic waste into valuable materials. This study highlights that the PE-degrading capability of the *K. cowanii* JNU01 strain could be pivotal for advancing methods to effectively biodegrade PE. In future research, we aim to conduct whole-genome sequencing and transcriptome analysis of *K. cowanii* JNU01 to investigate the mechanisms underlying PE biodegradation by characterizing the enzymes involved. This task presents challenges due to the potential existence of various unexpected pathways for PE biodegradation. Additionally, it is also essential to investigate the accessibility of PE into bacterial cells.

## Figures and Tables

**Table 1:** Microorganisms capable of biodegrading polyethylene (PE).

Microorganism	PE Type	Culture Conditions	Growth rate	FT-IR analysis	Metabolite analysis (MS)	Reference
*Kosakonia cowanii* JNU01	LDPE powder / film	28°C, Sole carbon source (PE), 35 days	OD_600_ = 0.62	Carboxylic acid, Hydroxyl group, Amide	Alkanes, ketones, and fatty acids	This study
*Enterobacter* sp. D1	LDPE film	37°C, Sole carbon source (PE), 31 days	OD_600_ = 0.24	Carbonyl bands, ether groups	Alkanes, alcohols, hydrocarbon, esters, and acids	[20]
*Acinetobacter* sp. NyZ450 & *Bacillus* sp. NyZ451	LDPE mulching film	28°C, Co-culture Sole carbon source (PE), 30 days	OD_600_ = 0.065	C=C bonds, O–H bonds (Hydroxyl group)	N/A	[34]
*Klebsiella pneumoniae* CH001	HDPE film	30 ± 2°C, Medium containing NB (Nutrient Broth), 2 months	N/A	Carboxylic group	carboxylic acids and alkanes	[42]
*Bacillus velezensis* C5	LDPE film	37°C, Sole carbon source (PE), 90 days	N/A	Alcohols, carbonyl bond, double bond, terminal double bond	n-alkanes (C_24_–C_29_)	[43]
*Pseudomonas aeruginosa* SKN1	LDPE powder	29°C, Sole carbon source (PE), 60 days	OD_600_ = approximat ely 0.24	C–H bond stretching, C–C bond stretching	N/A	[35]
*Bacillus cereus*, *Bacillus megaterium*, *Bacillus subtilis*	LDPE, LLDPE film	30 & 45°C, Co-culture Sole carbon source (PE), 90 days	N/A	Amide bond, hydroxyl bond,	Alkanes, alcohols	[23]
*Bacillus pumilus*, *Bacillus halodenitrificans*, *Bacillus cereus*	LDPE film	30°C, Sole carbon source (PE), 80 days	N/A	C=O stretch (ketone)	Ketones, alcohols, Alkanes	[44]

N/A: not applicable, LDPE: Low-Density Polyethylene, LLDPE: Linear Low-Density Polyethylene, HDPE: High-Density Polyethylene, MS: mass spectrometry

**Table 2:** Dissolved oxygen (DO) levels in PE media inoculated with *K. cowanii* JNU01.

Bacterium	Initial DO (mg/l)	DO after 1 day (mg/l)	DO after 2 days (mg/l)	DO after 4 days (mg/l)
*K. cowanii* JNU01	8.93 ± 0.01	2.79 ± 0.09	0.54 ± 0.02	0.27 ± 0.10

**Table 3:** Metabolites of polyethylene (PE) in PE media-inoculated with *K. cowanii* JNU01.

Number	Retention time (min)	Similarity (%)	Compound name	Molecular weight	Formula
1	29.263	96	2-Hexadecanone	240	C_16_H_32_O
2	30.940	80	9-Heptadecanone	254	C_17_H_34_O
3	31.100	94	Octadecane	254	C_18_H_38_
4	32.113	93	n-Hexadecanoic acid	256	C_16_H_32_O_2_
5	32.883	90	2-Heptadecanone	254	C_17_H_34_O
6	33.203	90	Octadecanal	268	C_18_H_36_O
7	34.127	85	6,10,14-Trimethylpentadecan-2-one	268	C_18_H_36_O
8	34.363	82	9-Octadecanone	268	C_18_H_36_O
9	34.493	95	Eicosane	282	C_20_H_42_
10	35.440	89	Octadecanoic acid	284	C_18_H_36_O_2_
11	36.190	84	2-Nonadecanone	282	C_19_H_38_O
12	37.503	82	10-Nonadecanone	282	C_19_H_38_O
13	37.607	95	Pentacosane	352	C_25_H_52_
14	38.497	90	2-Methylhexacosane	380	C_27_H_56_
15	39.217	82	2-Nonacosanone	422	C_29_H_58_O
16	40.053	85	Nonacosan-14-one	422	C_29_H_58_O
17	40.473	93	Dotriacontane	450	C_32_H_66_
18	41.997	91	2-Tritriacontanone	478	C_33_H_66_O

**Table 4:** FT-IR analysis results of PE powder cultured with *K. cowanii* JNU01.

Number	Wavenumber (cm^–1^)	Bond	Functional group	Change
[1]	1,000–1,200	C-O	Carboxylic acid	Increase
[2]	1,200–1,350	C-N	Amide	Increase
[3]	1,445–1,485	C-H	Alkyl	Decrease
[4]	2,850–3,000	C-H	Alkyl	Decrease
[5]	3,200–3,400	C-OH	Hydroxyl group	Increase
